# PDA Indolylmaleimides Induce Anti-Tumor Effects in Prostate Carcinoma Cell Lines Through Mitotic Death

**DOI:** 10.3389/fvets.2020.558135

**Published:** 2021-01-20

**Authors:** Jan Torben Schille, Ingo Nolte, Julia Beck, Daria Jilani, Catrin Roolf, Anahit Pews-Davtyan, Arndt Rolfs, Larissa Henze, Matthias Beller, Bertram Brenig, Christian Junghanss, Ekkehard Schütz, Hugo Murua Escobar

**Affiliations:** ^1^Department of Medicine, Clinic III–Hematology, Oncology, Palliative Medicine, University of Rostock, Rostock, Germany; ^2^Small Animal Clinic, University of Veterinary Medicine Hannover, Hannover, Germany; ^3^Chronix Biomedical, Göttingen, Germany; ^4^Leibniz-Institute for Catalysis, University of Rostock, Rostock, Germany; ^5^Centogene AG, Rostock, Germany; ^6^Institute of Veterinary Medicine, University of Göttingen, Göttingen, Germany

**Keywords:** mitotic death, mitotic slippage, whole transcriptome sequencing, human, dog, prostate cancer

## Abstract

Castrate resistant prostate cancer in men shares several characteristics with canine prostate cancer (PCa). Due to current insufficient therapies, evaluating novel therapeutic agents for late-stage PCa is of considerable interest for both species. PDA indolylmaleimides showed anticancer effects in several neoplastic cell lines. Herein, a comparative characterization of PDA-66 and PDA-377 mediated effects was performed in human and canine PCa cell lines, which is also the first detailed characterization of these agents on cells derived from solid tumors in general. While PDA-377 showed only weak growth inhibition on human PCa cell lines, PDA-66 inhibited proliferation and induced apoptosis in human and canine cell lines with concentrations in the low micromolar range. Morphological characterization and whole transcriptome sequencing revealed that PDA-66 induces mitotic death through its microtubule-depolymerizing ability. PDA-66 appears to be a worthwhile anti-mitotic agent for further evaluation. The similarities in cellular and molecular response observed in the cell lines of both origins form a solid basis for the use of canine PCa *in vivo* models to gain valuable interchangeable data to the advantage of both species.

## Introduction

Prostate cancer (PCa) is the most common malignancy diagnosed among males in almost all western countries (1). PCa emerges in an androgen-dependent or androgen-independent manner with a highly heterogeneous clinical course. Localized PCa has a 5-year survival rate close to 100% due to the availability of a broad range of curative treatment options. However, up to 20% of patients develops incurable and lethal metastatic castrate-resistant PCa within 5 years of follow-up. The development of novel therapeutic options for treating high-risk locally advanced PCa is needed (2–5).

Canine tumors are valuable naturally occurring models helping to reveal mechanisms in cancer development, behavior and treatment (6, 7). Prostatic neoplasia in dogs are reported with a prevalence below 1% (8) and poor prognosis (9–11). However, unlike in men there is no screening for early detection and only late stage cases are diagnosed. Actual numbers are likely to be much higher based on the high frequency of preneoplastic lesions (12). Canine prostate adenocarcinomas share several characteristics with human castrate-resistant PCa, e.g., increased occurrence with age, aggressive tumor progression, similar metastatic spread (lumbar spine and pelvis as well as lymph nodes), and castration-resistance (13, 14). Therefore, identifying and evaluating novel effective agents to treat late-stage, metastatic PCa may be beneficial for both species.

Indolylmaleimides as PDA derivatives PDA-66 and PDA-377 are synthetic molecules characterized by the conjugation of a maleimide compound with a bicyclic indole ring (15, 16). PDA-66 induces mitotic arrest and apoptosis in neuroblastoma, lung cancer, and canine lymphoma cells (15, 17), and has been shown to depolymerize microtubules (15). Known microtubule-destabilizing agents, such as *Vinca* alkaloids, are used in various cancers (18). These antimitotic drugs lead to failures in spindle formation and chromosome segregation in dividing cells, which activates the spindle assembly checkpoint leading to mitotic arrest. Prolonged mitotic arrest eventually triggers mitotic death (MD), an intrinsic form of regulated cell death (19, 20). MD is considered an onco-suppressive mechanism controlling mitotic failures and therefore prevents aneuploidy. Those failures include extensive DNA damage preventing replication, problems with the mitotic machinery (e.g., equal distribution of chromosomes) or failure of mitotic checkpoints leading to premature progress in the cell cycle (19, 21, 22).

A comprehensive characterization of PDA effects in human and canine PCa cells is missing. Before introducing such novel inhibitors into clinics, conducting an evaluation of these agents in model organisms is a prerequisite. Dogs classify as an extraordinary naturally occurring model for human PCa trials. As therapeutic options for dogs are limited and their metabolism is highly comparable to humans, clinical trials in dogs are considered to be of significant value (23). However, before addressing veterinary patients in trials evaluating novel agents, a detailed characterization of its effects *in vitro* is necessary.

Therefore, the aim of this study was to comparatively characterize the influence of PDA-66 and PDA-377 on two human prostate carcinoma cell lines, PC-3 and LNCaP, and on the canine cell line CT1258, which is also the first detailed characterization of these agents on cells derived from solid tumors. Besides cellular analysis, whole transcriptome sequencing was performed. Based on these results, canine PCa is evaluated as a model for clinical trials, accelerating the translation into human patients and providing direct benefit to both species.

## Materials and Methods

### Prostate Carcinoma Cell Lines and Cultivation

Two human and one canine prostate carcinoma cell line were used. The human PC-3 cell line (24) (DSMZ Cat# ACC-465, RRID:CVCL_0035) was cultivated in DMEM/Ham's F-12 medium (Biochrom GmbH, Berlin, Germany). The human LNCaP cell line (25) (DSMZ Cat# ACC-256, RRID:CVCL_0395) was grown in RPMI 1640 medium (Biochrom GmbH). The canine cell line TihoDProAdcarc1258 (26) (CT1258, RRID:CVCL_W737) was established by our group and cultivated in medium 199 (Live Technologies GmbH, Darmstadt, Germany). All media were supplemented with 10% heat-inactivated fetal bovine serum (FBS Superior, Biochrom GmbH) and 2% penicillin/streptomycin (Biochrom GmbH). All cells were cultivated at 37°C in a humidified atmosphere of 5% CO_2_.

### Indolylmaleimides PDA-66 and PDA-377

Synthesis and chemical structures of indolylmaleimides were previously described (15, 16, 27, 28). Both PDA derivatives were dissolved in dimethyl sulfoxide (DMSO, AppliChem GmbH, Darmstadt, Germany), and the stock solutions (10 mM) were stored at −20°C. For experimental use, the PDA dilutions were freshly prepared from the stock solution.

Different PDA concentrations and incubation times were used and compared with DMSO-exposed controls, as the PDA agents themselves were dissolved in DMSO. The final DMSO concentrations of the control samples were equivalent to the highest DMSO doses in the PDA-treated samples to ensure that no possible effects of the solvent were measured.

### Live Cell Imaging

PC-3, LNCaP and CT1258 cells were seeded in 96-well plates with a cell density of 1 × 10^4^ cells per well and incubated overnight in 150 μl of culture medium. After 24 h different concentrations of the PDA derivatives (0.25–10 μM) were applied (0.1% DMSO as control). The cells were observed for 72 h in 150 μl incubation medium under a live cell imaging microscope (DMI 6000 B, Leica Mikrosysteme Vertrieb GmbH, Wetzlar, Germany) at 37°C with 5% CO_2_. An image of each well was captured every 15 min during the incubation period and these single images were combined to create time-lapse movies.

### Analysis of Morphology

Morphological changes mediated by PDA were analyzed by May-Grünwald-Giemsa staining.

Microscope slides were placed in 60 cm^2^ cell culture dishes and covered with 10 ml of culture medium. Per dish and per cell line, 1 x 10^6^ cells were seeded. On the following day, the microscope slides were transferred to new dishes with incubation medium or 0.15% DMSO for the control cells. The slides were exposed to the PDA agents in two different settings. For the first setting, the slides were incubated with 15 μM PDA-66 or PDA-377 for 24 h, an application equal to the cells used in the transcriptomic analyses. For the second setting, the slides were incubated with 5 μM PDA-66 or PDA-377 for 72 h, a concentration that showed moderate effects in all cell lines in live cell imaging. Microscope slides were washed with PBS and air dried at room temperature. The slides were stained in May-Grünwald's eosine-methylene blue solution (Merck KGaA, Darmstadt, Germany) (undiluted) for 5 min and rinsed with tap water. Afterwards, they were stained for 15 min in Giemsa's azur eosin methylene blue solution (Merck KGaA) (1:10 dilution) and rinsed thoroughly with tap water again. The slides were left to air dry before analysis.

### BrdU Proliferation Assay

Proliferative index of cells in response to PDA-66 and PDA-377 exposure was evaluated using the assay Cell Proliferation ELISA, BrdU (colorimetric) (Roche Diagnostics Deutschland GmbH, Mannheim, Germany). The assay measures the incorporation of the thymidine analog 5-bromo-2-deoxyuridine (BrdU) into newly synthesized DNA of replicating cells using an anti-BrdU monoclonal antibody.

All three cell lines were seeded in 96-well plates with a density of 1 × 10^4^ cells per well and incubated in 150 μl culture medium for 24 h to allow the cells to attach to the surface. Afterwards, the cells were exposed to different concentrations of the PDA derivatives. The human cell lines were treated with 0.25–10 μM of the derivatives and 0.1% (v/v) DMSO as control. CT1258 cells were exposed to 0.25–25 μM of both derivatives (0.25% DMSO as control) as no significant effect was displayed after 24 h with 10 μM PDA-66. The proliferation assay was carried out in accordance with the manufacturer's protocol for adherent cells with the exception that BrdU was added simultaneously with the PDAs. The reaction products were quantified by measuring the absorbance at 370 nm minus the absorbance at 492 nm over a 30 min period using the Multi-Mode Reader Synergy 2 (BioTek Instruments Inc., Winooski, VT, USA). Each single experiment was performed 8-fold.

After this point, no further tests were performed with PDA-377 due to its limited effect on the human cell lines.

### Cell Count Analyses

The concentrations used for cell count analysis and apoptosis are based on the 72 h values of the BrdU assay. Concentrations were chosen, that lead to 50% inhibition (2.5 μM PDA-66 in PC-3 and 5 μM PDA-66 in LNCaP). These values were chosen to ensure that there would be some margin in both directions to calculate possible significances, even if the other tests would show higher or lower efficiencies. Based on this criterion, 5 μM PDA-66 should have been used for the CT1258 cell line. However, this concentration showed no effect after 24 h. As it was crucial to be able to detect effects early after exposure for the transcriptomic analysis, 15 μM was used for this cell line, the first concentration to show a significant effect in the BrdU assay after 24 h of exposure.

Cells were seeded in 24-well plates with a cell density of 5 × 10^4^ cells per well in 1 ml culture medium and incubated overnight. Based on the proliferation assay, PC-3 cells were treated with 2.5 μM PDA-66 and 0.025% DMSO, LNCaP with 5 μM PDA-66 (0.05% DMSO), and CT1258 cells with 15 μM PDA-66 (0.15% DMSO). After 24 h, 48 h and 72 h, the cells were detached by TrypLE™ Express (Thermo Fisher Scientific Inc., Waltham, MA, USA) and counted via automatic cell counter (CellometerTM Auto T4, Nexcelom Bioscience LLC, Lawrence, MA, USA). The experiment was carried out in biological replicates three times.

### Analyses of Early and Late Stage Apoptosis

Apoptosis rates were determined by staining cells with the fluorescence dyes Annexin V FITC and Propidium Iodide (PI) (Annexin V-FITC Detection Kit, PromoCell GmbH, Heidelberg, Germany) in accordance with the manufacturer's protocol and determined by flow cytometry using a FACSCaliburTM (BD Biosciences GmbH, Heidelberg, Germany). In addition to the cell specific concentrations used in the cell count analysis, two lower concentrations were used to demonstrate that the induction of apoptosis already starts at lower concentrations. The data were evaluated using BD CellQuest software (BD CellQuest Pro, RRID:SCR_014489).

All three cell lines were seeded in 12-well plates with a cell density of 2 × 10^5^ cells per well and incubated in 1.5 ml culture medium overnight and exposed after 24 h to three different PDA-66 concentrations and DMSO based on the BrdU assay and cell count analyses. PC-3 cells were exposed to 0.5, 1.0, and 2.5 μM PDA-66 (0.025% DMSO as reference), LNCaP to 1.0, 2.5, and 5.0 μM PDA-66 (0.05% DMSO), and CT1258 to 7.5, 10 and 15 μM PDA-66 (0.15% DMSO). After the drug exposure period, the medium was saved to collect the non-adherent cell fraction to count all treated cells regardless of their condition. Remaining adherent cells were detached by TrypLE™ Express (Thermo Fisher Scientific Inc, Waltham, MA, USA) and combined with the medium cell fraction. In addition to the double stained samples, for each cell line and each incubation time point, single stained controls were measured. Exposure of the cells to PDA-66 and DMSO as reference was conducted in biological replicates three times.

### Transcriptomic Analyses

As PDA-66 is a strong inducer of apoptosis, the transcriptomic analysis was performed on early time points (12 and 24 h). This early time points, before all the apoptosis signaling cascades are fully active and most transcripts would be cell death related, were chosen for the best chance to detect the direct effects of PDA exposure. As 15 μM was the first concentration to show significant effects in CT1258 after 24 h, this concentration was used for all cell lines to keep the transcriptomic data comparable (0.15% DMSO for the control groups). After 12 and 24 h, the cells were harvested and total RNA was extracted using the AllPrep DNA/RNA Mini Kit (Qiagen GmbH, Hilden, Germany) in accordance with the manufacturer's protocol. For each treatment condition (DMSO control and PDA-66 exposed cells), three independent biological replicates were prepared and sequenced.

RNA integrity numbers (RIN) were determined using a Bioanalyzer 2100 (Agilent Technologies Inc., Santa Clara, CA, USA). Sequencing libraries were prepared using 1 μg total RNA with RIN > 7. PolyA RNA was enriched and ligated to sequencing adapters using the NEBNext Ultra RNA preparation kit (New England Biolabs Inc., Ipswich, MA, USA) in accordance with the manufacturer's protocols. Single-read sequencing (75 bp) was conducted on an Illumina NextSeq500 (Illumina Inc., San Diego, CA, USA). Sequences were aligned to the canine genome (Broad CanFam3.1/canFam3, Sep. 2011) using the Burrows-Wheeler Aligner (BWA) (29) (BWA, RRID:SCR_010910). For each of the 24,581 annotated protein-coding canine genes (EMBL gene ID nomenclature), the aligned reads were counted using the R package GAGE (30) (GAGE, RRID:SCR_017067). For the human PC-3 and LNCaP, mapping and read counting were conducted using the RNA Express (version: 1.0.0) workflow within the Illumina basespace environment (BaseSpace, RRID:SCR_011881). In brief, after mapping to the human HG19 reference genome using the STAR (2.3.1s) aligner (STAR, RRID:SCR_015899) read counts for 23,710 annotated RefSeq genes were generated.

### Statistical Analyses

Within each experiment, results were described using the mean values of the replicates. The graphs show the mean ± standard deviation. Significant differences between treatment and control were calculated by Dunnett's multiple comparison test for the confidence levels 95, 99, and 99.9% (BrdU proliferation assay and analyses of apoptosis) or student's *t*-test (cell count analyses) using SAS enterprise guide 7.1 (Statistical Analysis System, RRID:SCR_008567). Differences were considered statistically significant for *p* < 0.05. IC50 values were calculated using GraphPad Prism 7.02 (GraphPad Prism, RRID:SCR_002798).

Differential gene expression analyses for canine and human samples were conducted using the BioconductorR package edgeR 3.14.0 (31) (edgeR, RRID:SCR_012802). The average total read counts mapped within annotated genes were 12.2M (STD: 0.7M), 15.8M (STD: 1.1M), 13.5M (STD: 0.9M) for CT1258, PC-3 and LNCaP samples, respectively. Each PDA-66 treatment group (12 or 24 h) was compared to the respective control cells treated with DMSO. After multidimensional scaling and plotting of the data, one PC-3 DMSO treated control sample was identified as outlier and was excluded from further analyses so that the PC-3-24 h-DMSO group consisted of only two independent samples. Genes with a false discovery rate adjusted *p*-value (FDR) of < 0.001 were considered to have significantly different expressions compared to the control samples. These genes were mapped to the Kyoto Encyclopedia of Genes and Genomes (KEGG) pathways for functional enrichment analyses using the Database for Annotation, Visualization and Integrated Discovery (DAVID) Functional Annotation Tool (32, 33) (DAVID, RRID:SCR_001881).

## Results

### PDA-66 and PDA-377 Cause Morphological Changes

**Figure 1:**
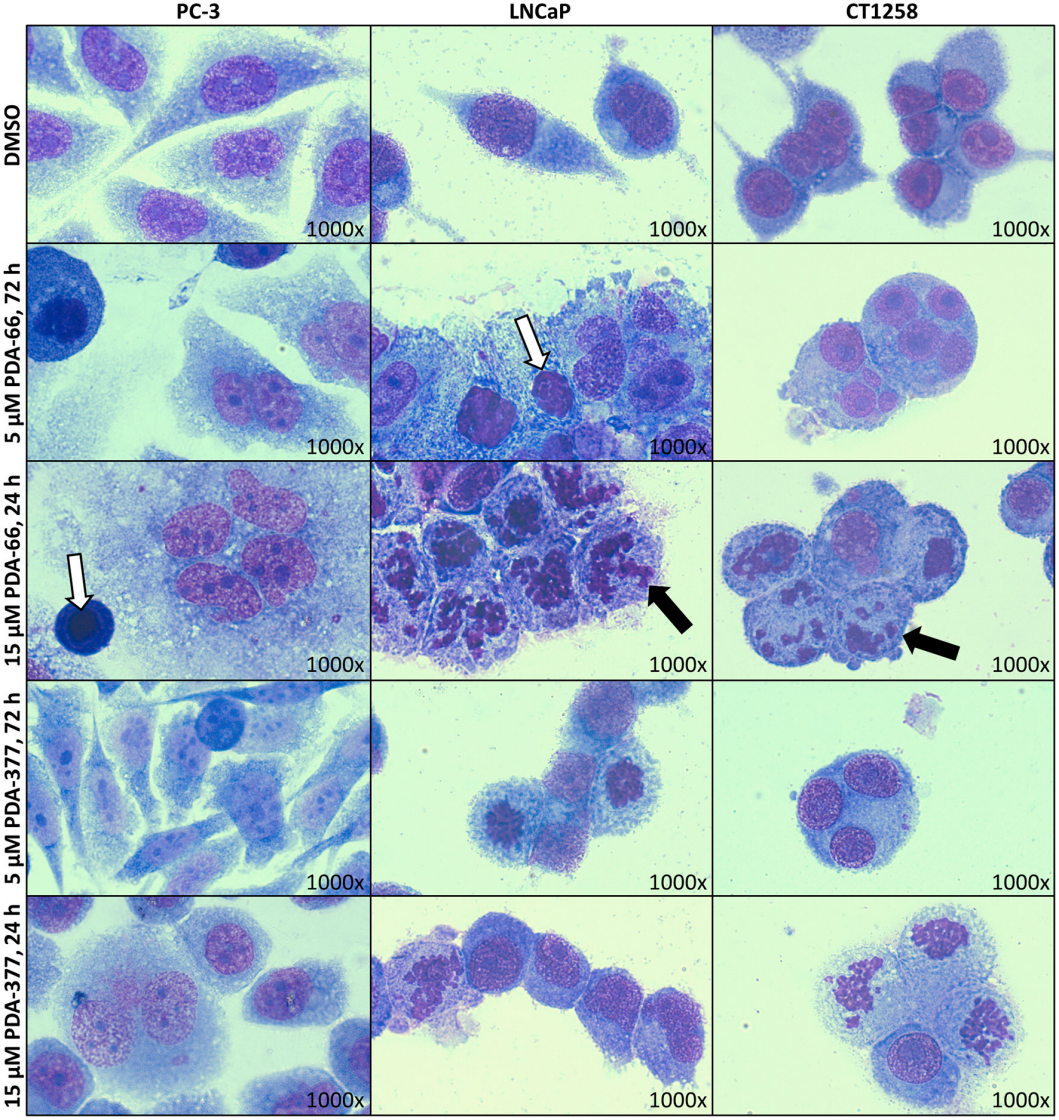
PC-3, LNCaP, and CT1258 cells were grown on microscope slides and incubated with both PDA derivatives for either 72 h with 5 μM PDA or 24 h with 15 μM. Representative pictures are displayed. After incubation with PDA-66, cells tend to aggregate and the cytoplasmic border loosens. Multinucleated cells, pyknosis (white arrows) and karyorrhexis (black arrows) can be determined. Post PDA-377 application, only minor morphological changes can be observed in PC-3 cells. In LNCaP and CT1258 cells, the reactions of cytoplasm and nucleus are similar to incubation with PDA-66.

May-Grünwald-Giemsa staining revealed morphological changes in the three tested cell lines (Figure 1). Incubation with 5 μM PDA-66 caused an increased amount of large multinucleated cells as well as cells with pyknotic nuclei and karyorrhexis. Moreover, the cells aggregated in clusters and their cytoplasmic borders loosened. In LNCaP and CT1258 cells, 24 h after high-dose (15 μM) PDA-66 application, karyorrhexis was pronounced in most cells.

PDA-377 incubation caused no visual effects after 5 μM treatment for 72 h, and only minor effects on the PC-3 cell line after 15 μM treatment for 24 h. The morphological changes on LNCaP and CT1258 cells were similar to PDA-66 incubation but less pronounced.

**Figure 2:**
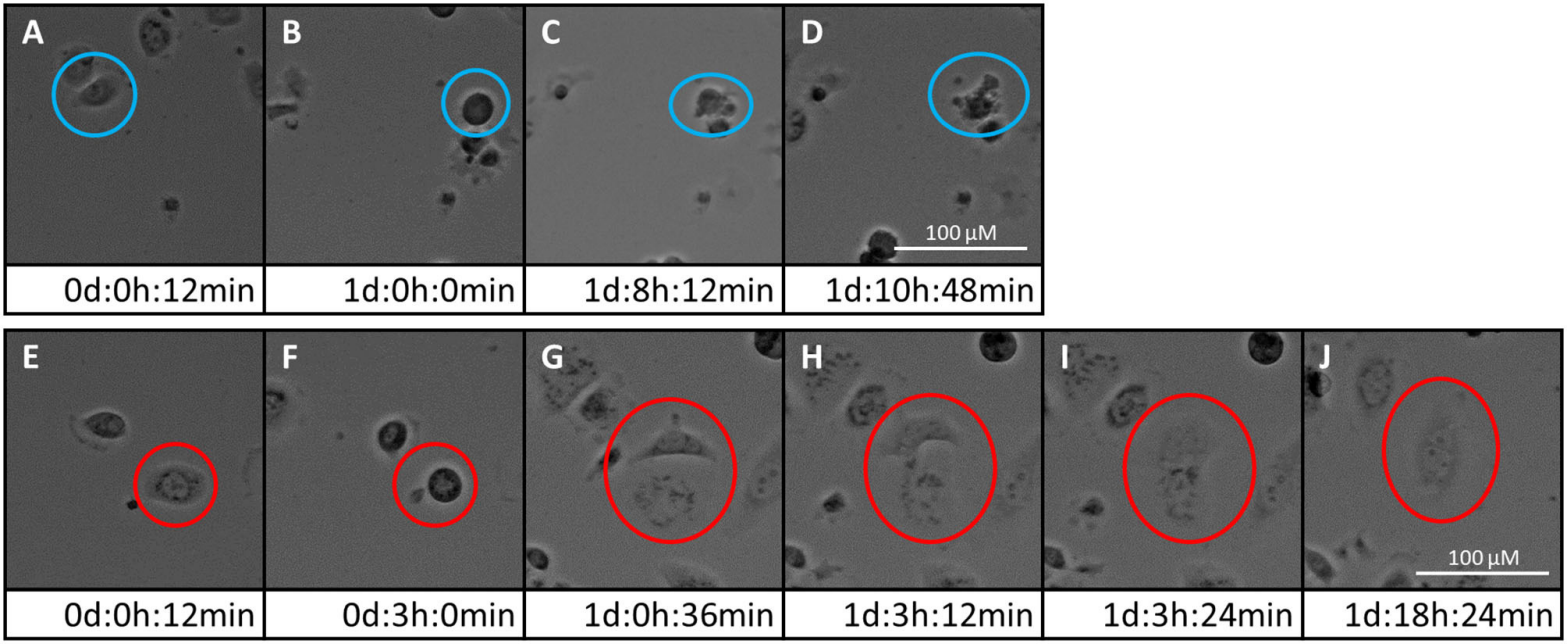
Two fates of PC-3 cells exposed to 5 μM PDA-66. Live cell imaging pictures show the same cell and image section during a 72 h incubation period. **(A–D)** apoptosis of cell in blue circle; **(A)** start of live cell imaging; diploid cell; **(B)** cell becomes round/detached in preparation for cell division; **(C)** formation of apoptotic bodies; **(D)** dead cell; **(E–J)** mitotic slippage of cell in red circle; **(E)** start of live cell imaging; diploid cell; **(F)** cell becomes round/detached in preparation for cell division; **(G)** two daughter cells with unbalanced distribution of chromosomes; **(H,I)** daughter cells merge again; **(J)** survival of a tetraploid/multi-nucleated cell. Please see Supplementary Material for the full movie.

Time-lapse imaging movies of the three PCa cell lines showing the cellular response to 5 and 10 μM of either PDA-66 or PDA-377 as well as the control groups are given for demonstration (Supplementary Movies 1–15). Moreover, the formation of enlarged multi-nucleated cells as well as apoptosis during or shortly after mitosis was observed at higher PDA concentrations (Figure 2).

### PDA-66 and PDA-377 Inhibit Proliferation of Prostate Carcinoma Cell Lines

**Figure 3:**
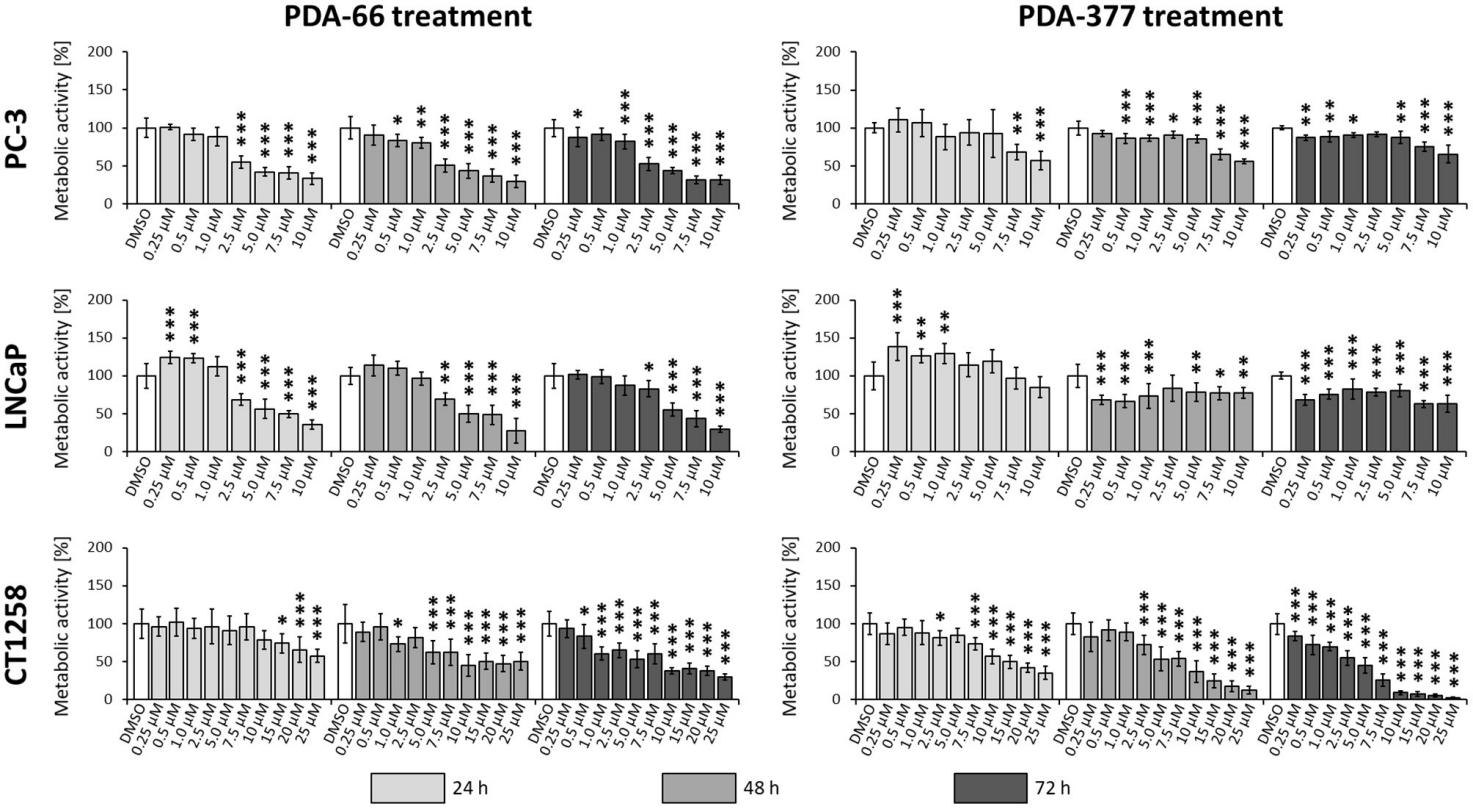
PC-3, LNCaP and CT1258 cells were exposed to increasing concentrations of PDA-66 and PDA-377 ranging from 0.25 to 10 μM and up to 25 μM in the case of CT1258. The cells were incubated for 24, 48, and 72 h, respectively. BrdU proliferation assay was used to determine the proliferative index. The results are expressed as a percentage of the DMSO-exposed control cells. The diagrams show the mean ± standard deviation of eight measurements. Significance of a treatment effect compared to the respective DMSO control (white bar) was determined using Dunnett's Multiple Comparison Test. *, *p* < 0.05; **, *p* < 0.01; ***, *p* < 0.001.

PDA-66 exposure demonstrated an inhibitory effect on both human cell lines and the canine cell line determined by BrdU proliferation assay (Figure 3). Application of 2.5 μM PDA-66 resulted in a significant decrease in the proliferative index in PC-3 and LNCaP cells 24 h after treatment. Moreover, PC-3 cells displayed significantly slower proliferation at 1.0 μM PDA-66 exposure after 48 and 72 h. Proliferation was reduced to 50% compared to the DMSO-exposed controls at 2.5 μM PDA-66 for PC-3 and at 5.0 μM for LNCaP cells after 72 h. Compared to the human cell lines, inhibition of proliferation of the canine cell line CT1258 was delayed, but was also more pronounced after 72 h post PDA-66 application, starting with concentrations of 0.5 μM. For all cell lines, the proliferative index did not drop below 25%. IC50 values were 2.07 μM (PC-3), 4.23 μM (LNCaP), and 2.19 μM (canine cell line CT1258) after a 72 h incubation period.

Incubation with PDA-377 also led to a significant decrease in the proliferative index in all tested cell lines. PC-3 and LNCaP cells were inhibited at concentrations of 0.5 μM and 0.25 μM PDA-377 after 48 h. However, proliferation did not drop below 50% compared to the control samples for all tested incubation times and doses in the two human cell lines. For the canine cell line CT1258, the proliferative index dropped below 10% with an IC50 of 3.14 μM after a 72 h incubation period. Compared to PDA-66, PDA-377 treatment displayed a weaker inhibition on the human cell lines, especially within the first 24 h and a slightly stronger effect on the canine PCa cell line.

### PDA-66 Induces Apoptosis

**Figure 4:**
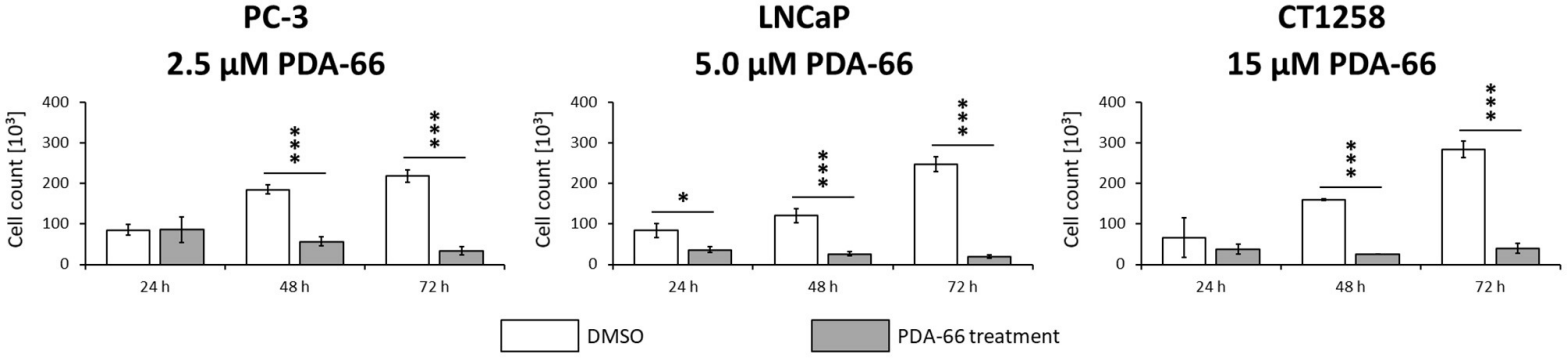
PC-3 cells were incubated with 2.5 μM PDA-66, LNCaP cells with 5 μM PDA-66 and CT1258 cells with 15 μM PDA-66 for 24, 48, and 72 h. After the incubation period, cells were counted via automatic cell counter. The diagrams show the mean ± standard deviation of three independent counting experiments. Significance of a treatment effect compared to the DMSO control was determined using the student's *t*-test. *, *p* < 0.05; ***, *p* < 0.001.

Consistent with live cell imaging observations and the proliferation assay, a significant change in the total number of cells was observed 48 h after PDA-66 application in all tested cell lines compared to their tested control cells (Figure 4). While the control cells showed a steady increase in cell numbers over time and at least doubled within 72 h, the number of PDA-66 treated cells decreased. The decrease below the amount of seeded cells (5 × 10^4^) indicated the induction of cell death.

**Figure 5:**
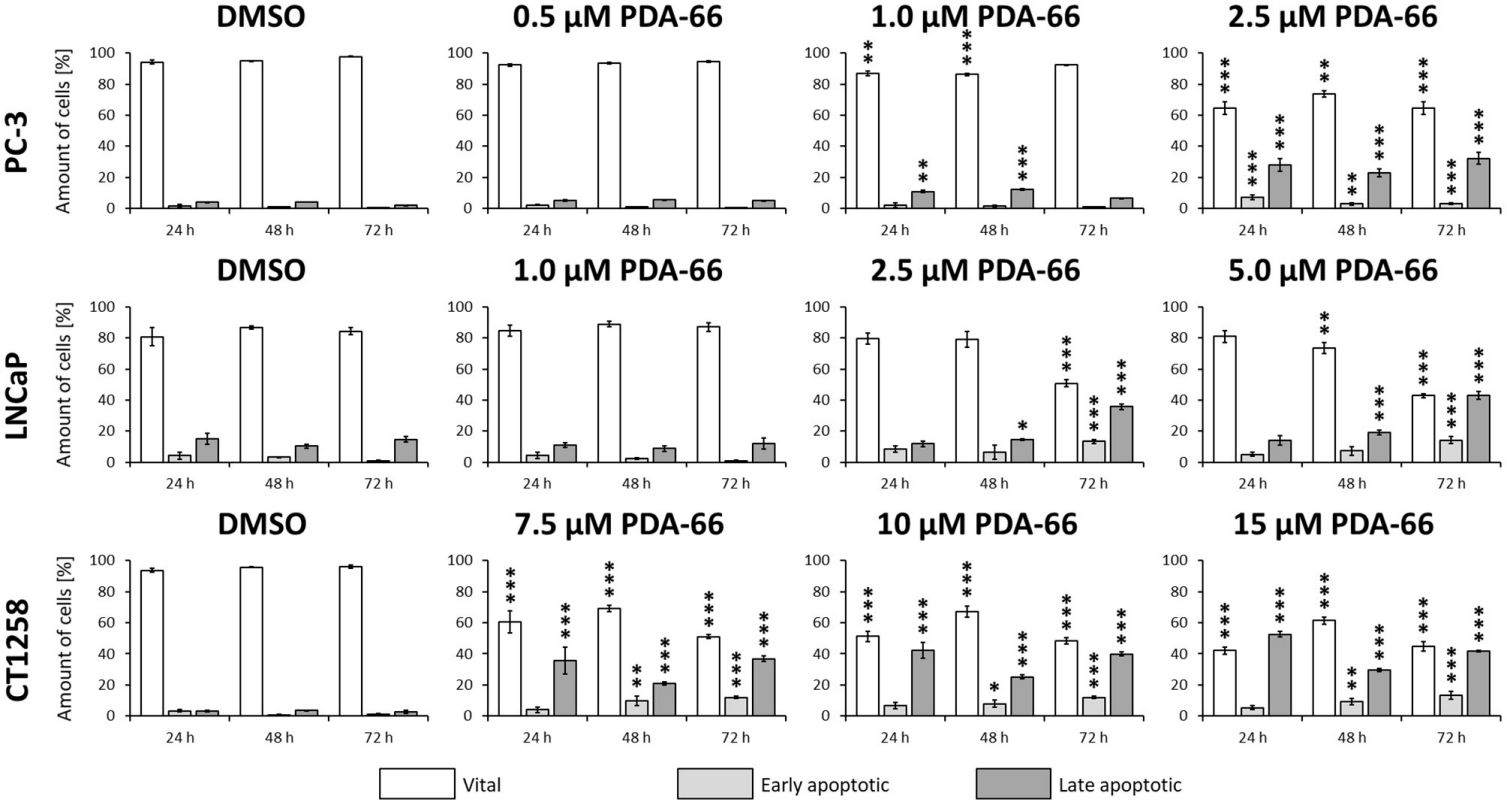
PC-3 cells were exposed to 0.5, 1.0, and 2.5 μM PDA-66, LNCaP cells were exposed to 1.0, 2.5, and 5.0 μM PDA-66, and CT1258 cells were exposed to 7.5, 10, and 15 μM PDA-66 for 24, 48, and 72 h, respectively. As a reference, DMSO-exposed cells were analyzed. Analysis of apoptosis was performed using Annexin V-FITC and propidium iodide (PI) staining with subsequent flow cytometry analysis. Rates of vital (FITC-, PI-) early apoptotic (FITC+, PI-) and late apoptotic (FITC+/-, PI+) cells are displayed as the mean ± standard deviation of three independent measurements. Significances compared to the DMSO control were determined using Dunnett's multiple comparison test. *, *p* < 0.05; **, *p* < 0.01; ***, *p* < 0.001.

PDA-66 significantly induced apoptosis in all three PCa cell lines compared to the DMSO-exposed controls at different concentrations (Figure 5, representative dot blots are given in Supplementary Figure 1). Application of 2.5 μM PDA-66 led to an increase in both early and late apoptotic cells in the two human cell lines after 72 h, reaching up to 35.8%. The rate of late apoptotic/dead cells even increased to 43% in LNCaP cells after incubation with 5 μM PDA-66. In the canine cell line, early apoptotic induction increased over time for all concentrations up to 13.4% (72 h, 15 μM PDA-66), while the rate of late apoptotic/dead cells varied between 20.9 and 52.7%.

### Transcriptomic Analyses of PDA-66 Treated Prostate Carcinoma Cells

**Figure 6:**
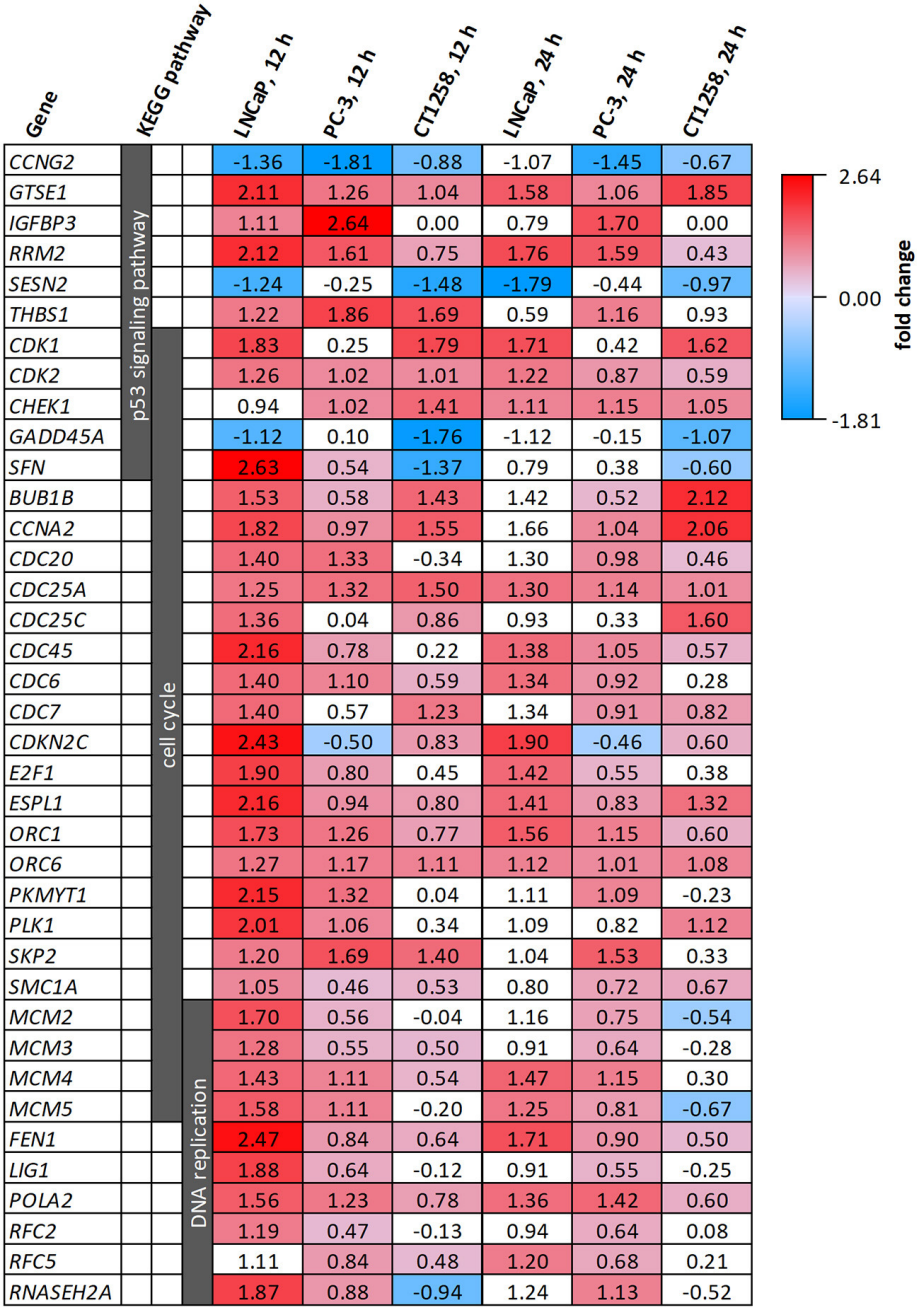
DEGs belonging to the KEGG pathways p53 signaling pathway, cell cycle and DNA replication after PDA-66 exposure. Numbers given for each gene are the fold changes expressed as logarithmized ratios (base 2) in the PCa cell lines after 12 and 24 h. Values highlighted in red or blue indicate significantly higher or lower expression compared to the DMSO control. Non-highlighted values were not significantly different (FDR ≥ 0.001).

All cell lines displayed a higher number of differentially expressed genes (DEG; FDR < 0.001) after 12 h compared to the 24 h treatment groups (Table 1), correlating with larger distances between clusters of the multidimensional scaling (MDS) plots and the control samples (Supplementary Figure 2). PC-3 and CT1258 cells showed more than 4,000 DEG after a 12 h and 24 h incubation period with PDA-66. In the LNCaP cell line, there were only up to 479 DEGs. Therefore, we focused on LNCaP for the KEGG pathway analysis (Table 2) with corresponding PC-3 and CT1258 values in comparison. Cell cycle, DNA replication and p53 signaling pathway were the only KEGG pathways significantly enriched (FDR < 0.05) in all three cell lines. Within these three pathways, 38 genes showed at least three significant values compared to the corresponding controls for the six analyzed groups and were further analyzed (Figure 6). The fold change pattern was highly consistent between the six groups. Within the relevant 38 genes, only five genes were inconsistent in the direction of their deregulation (upregulated vs. downregulated) across the three cell lines.

**Table 1:** Number of differentially expressed genes (DEGs) in treated prostate carcinoma cell lines compared to control samples.

**Cell line**	**Application**	**DEGs**
PC-3	15 μM PDA-66, 12 h	5,198
LNCaP	15 μM PDA-66, 12 h	479
CT1258	15 μM PDA-66, 12 h	5,316
PC-3	15 μM PDA-66, 24 h	4,013
LNCaP	15 μM PDA-66, 24 h	205
CT1258	15 μM PDA-66, 24 h	4,289

**Table 2:** KEGG pathways in LNCaP cells with an FDR < 0.05 and the corresponding values of these pathways in PC-3 and CT1258 cells after 12 h **(A)** and 24 h **(B)** of PDA-66 exposure.

**(A)**	**LNCaP**		**PC-3**		**CT1258**	
**KEGG pathways, 12 h PDA-66**	**Genes**	**FDR**	**Genes**	**FDR**	**Genes**	**FDR**
Cell cycle	29	4.99E-18	54	8.79E-04	69	2.87E-09
DNA replication	14	3.29E-11	23	1.82E-04	22	1.99E-03
p53 signaling pathway	10	7.24E-04	30	2.98E-02	38	1.49E-05
Fanconi anemia pathway	9	8.06E-04	23	1.60E-01	32	8.51E-06
Retinol metabolism	8	1.88E-02				
Base excision repair	6	1.96E-02	16	1.84E-01	20	8.62E-03
Pyrimidine metabolism	10	2.00E-02	35	7.51E-01	44	3.77E-03
Progesterone-mediated oocyte maturation	9	2.46E-02			45	7.43E-05
Mismatch repair	5	3.46E-02	11	5.62E-01	17	6.13E-04
**(B)**	**LNCaP**		**PC-3**		**CT1258**	
**KEGG pathways, 24 h PDA-66**	**Genes**	**FDR**	**Genes**	**FDR**	**Genes**	**FDR**
Cell cycle	14	6.44E-08	46	1.16E-03	54	1.01E-11
Retinol metabolism	7	2.18E-03				
DNA replication	5	1.36E-02	24	5.55E-07		
p53 signaling pathway	6	2.02E-02	21	5.88E-01	27	4.16E-05
Metabolism of xenobiotics by cytochrome P450	6	3.12E-02				
Chemical carcinogenesis	6	4.35E-02				

*Missing values showed no enrichment of genes for this pathway*.

## Discussion

In the present study, the effects of PDA-66 and PDA-377, two indolylmaleimide derivatives, were comparatively evaluated on three prostate carcinoma cell lines with a diverse genetic background. The human PC-3 and LNCaP cells are among the most used and best described PCa cell lines (34, 35), both established from metastatic adenocarcinomas. While PC-3 is androgen-independent (36), LNCaP is described as androgen-sensitive (37, 38). Well-characterized canine PCa cell lines are rare. Herein, CT1258 was used, a canine cell line derived by our working group from a metastatic adenocarcinoma, which has been previously established and characterized (26, 39–42).

Both PDA derivatives caused two distinct morphological changes in all tested cell lines. Live cell imaging and May-Grünwald-Giemsa staining revealed clear apoptotic features through observed pyknosis and karyorrhexis in May-Grünwald-Giemsa staining as well as the formation of apoptotic bodies in live cell imaging. These features match the proposed mechanism of induced mitotic arrest and subsequent MD. Secondly, some enlarged multinucleated cells formed. Cells can overcome the mitotic arrest before MD is triggered; a process known as slippage. After DNA replication in the S phase and escaping the mitotic arrest, these cells fail cytokinesis and/or the equal distribution of chromosomes, leading to enlarged multi-nucleated cells similar to those we observed (22, 43, 44).

Proliferation was measured via BrdU assay to quantify growth inhibition seen in live cell imaging. PDA-66 caused significant and dose-dependent anti-proliferative effects in all tested PCa cell lines. The herein demonstrated doses to inhibit proliferation significantly by at least 50% were between 5 and 10 μM PDA-66 within 72 h. The analyzed PCa cell lines were slightly less sensitive compared to other neoplastic cell lines like human neuroblastoma, human acute lymphoblastic leukemia or canine lymphoma (15–17). Consistent with live cell imaging movies, a significant reduction in total cell count and induction of apoptosis were separately quantified after PDA-66 incubation in all cell lines.

PDA-377 also induced anti-proliferative effects in all tested cell lines. However, for both human cell lines, the inhibition of proliferation was limited and most likely dose-independent, as a comparable decrease in proliferation was measured for almost all tested concentrations after 48 h. The proliferative index did not drop below 50%. In the canine cell line, the proliferation inhibition was clearly dose-dependent. After a 72 h incubation period, the proliferative index dropped below 10% with an IC50 of 3.14 μM. PDA-377 may be suitable for the treatment of prostate cancer in dogs. Nevertheless, no further testing with this substance was conducted, as the limited effect on the human cell lines contradicts the cross-species approach with dogs as a model to generate transferable findings to the benefit of both species.

To confirm whether MD takes place, whole transcriptome sequencing was conducted. The transcriptome analysis revealed the KEGG pathways cell cycle, p53 signaling pathway and DNA replication to be the only pathways with an enrichment of genes across the three PCa cell lines exposed to PDA-66. Other known microtubule-depolymerizing agents, like *Vinca* alkaloids or colchicine-binding site drugs, lead to failures in mitotic spindle formation and therefore chromosome segregation, which triggers the spindle assembly checkpoint (SAC; mitotic checkpoint). SAC activation causes mitotic arrest in the metaphase of the M phase of the cell cycle. Within the enriched KEGG cell cycle pathway, several important genes involved in SAC are upregulated after PDA-66 exposure, e.g., *BUB1B, CDC20, CDK1*, and *MAD2*. These DEGs are commonly involved in MD (20, 21, 45, 46).

MD resembles intrinsic apoptosis, and p53 and its pathway play a major role in most cells undergoing MD (19, 21, 46). Little is known about the downstream targets of SAC that trigger MD following a prolonged mitotic arrest (20), but the upregulated cyclin-dependent kinases CDK1 and CDK2, normally known for cell cycle progression, are directly linked with apoptosis after stress events like a mitotic arrest (47, 48).

Interestingly, there was also an upregulation of genes involved in G1/S phase cell cycle progression and DNA replication, especially within the two human cell lines. Given the M phase cell cycle arrest induced by SAC, an enrichment of S phase transcripts is unexpected. Moreover, G1/S phase transcripts were downregulated in canine lymphoma cell lines after PDA-66 exposure (17). However, formation of enlarged multinuclear cells, as observed in the PCa cell lines, has not been described for these lymphoma cells post PDA-66 incubation. These multinuclear cells have three possible fates: Post-slippage cell death (before re-entering mitosis), senescence or further proliferation (43, 49, 50). Senescent polyploid cells, locked in the next interphase after slippage, might be an explanation for the enrichment of G1/S phase transcripts seen in the PCa cell lines after PDA-66 application. For example, tumor suppressor *BRCA1* was significantly upregulated compared to the controls in all PCa cell line samples post PDA-66 exposure (data not shown), a gene that plays an important role in interphase checkpoint activation (51).

The observed morphological changes and the transcriptome analysis match other anti-mitotic drugs causing MD. While most cells die as a result of this intrinsic cell death, some cells remain in a polyploid state after mitotic slippage. These cells are usually senescent and therefore incapable of further proliferation. However, they bear the risk of reinforcing the tumor, as their potential proliferation can lead to aneuploid cells, a hallmark of cancer (43, 49, 50, 52). The selection of aneuploid karyotypes promotes aneuploidy tolerance, a major driving force in cancer evolution and drug resistance (46, 52). Docetaxel, the first-line treatment for castration-resistant prostate cancer in men, is also an anti-mitotic drug causing microtubule perturbations. Similar to PDA-66, treating PC-3 cells with Docetaxel generates multinucleated polyploid cells, which have been linked to clinical relapse and chemoresistance (53). While slippage limits the effectiveness of anti-microtubule drugs, these substances have been proven successful in clinics and are commonly used in a variety of malignancies in addition to PCa (18, 20, 49, 50).

PCa cell lines of human and canine origin showed comparable cellular and molecular reactions, including significant inhibition of proliferation and induced apoptosis after PDA-66 incubation. Furthermore, efficacy of PDA-66 was not influenced by the androgen status of the cell line. As PDA-66 mediates its effects in a hormone-independent manner through microtubule destabilization, the compound is suitable to be used in androgen-dependent as well as -independent PCa subtypes. Based on the findings of this study, PDA-66 appears to be a worthwhile agent for further evaluation and potential treatment of PCa in dogs and, if successfully introduced, also in humans.

The cellular and molecular *in vitro* characterization of PDA-66 effects on PCa cells encourage, as a next step, to initiate an early clinical trial in dogs to evaluate the compound in naturally occurring PCa in the presence of an immune system. First, however, a pharmacokinetic study in dogs needs to be performed. An early study in mice revealed that *i.p*. application of 100 mg/kg PDA-66 once per day was well-tolerated (17). The value for humans will be enhanced by the fact that dogs have several advantages over laboratory models, e.g., mice for comparative pharmacokinetics, as dogs represent an outbred non-immunodeficient model population. As companion animals, dogs are exposed to the same environmental risk factors as humans and the parallel evolution of humans and dogs led to a more similar genomic organization compared to rodents (54–57). Treatment of spontaneously occurring PCa in dogs can provide additional valuable information that can benefit both humans and dogs.

## Data Availability Statement

The original contributions presented in the study are publicly available. This data can be found at: https://www.ncbi.nlm.nih.gov/geo/query/acc.cgi?acc=GSE162832.

## Author Contributions

JS carried out partial BrdU assay, May-Grünwald-Giemsa staining, cell treatment and RNA extraction for whole transcriptome analysis, performed data analysis, interpretation and visualization, and drafted the manuscript. IN provided the resources for all cellular analyses, supervised the study, and revised the manuscript. JB performed transcriptomic sequencing and revised the manuscript. DJ carried out partial BrdU assay, live cell imaging, cell count, and flow cytometry analyses. CR helped with primary study design. AP-D synthesized the PDA compounds. AR participated in initial compound generation. LH evaluated the May-Grünwald-Giemsa staining. MB supervised PDA compound design and synthesis. BB and ES provided the resources for the transcriptomic analysis and supervised the transcriptomic work packages. CJ and HM performed the primary study design. In addition, HM supervised the study and revised the manuscript. All authors contributed to the article and approved the submitted version.

## Conflict of Interest

The authors declare that the research was conducted in the absence of any commercial or financial relationships that could be construed as a potential conflict of interest. The handling editor declared a past co-authorship with one of the authors HM.

## Supplementary Material

Further supplementary videos can be requested from the corresponding author.

## Abbreviations

PCa: prostate cancer
MD: mitotic death
DEG: differentially expressed gene
SAC: spindle assembly checkpoint.

## References

[1] Bray F Ferlay J Soerjomataram I Siegel RL Torre LA Jemal A. Global cancer statistics 2018: globocan estimates of incidence and mortality worldwide for 36 cancers in 185 countries. CA Cancer J Clin. (2018) 68:394–424. 10.3322/caac.21492 30207593

[2] Kirby M Hirst C Crawford ED. Characterising the castration-resistant prostate cancer population: a systematic review. Int J Clin Prac. (2011) 65:1180–92. 10.1111/j.1742-1241.2011.02799.x 21995694

[3] Attard G Parker C Eeles RA Schroder F Tomlins SA Tannock I Prostate cancer. Lancet. (2016) 387:70–82. 10.1016/S0140-6736(14)61947-4 26074382

[4] Hoang DT Iczkowski KA Kilari D See W Nevalainen MT. Androgen receptor-dependent and -independent mechanisms driving prostate cancer progression: opportunities for therapeutic targeting from multiple angles. Oncotarget. (2017) 8:3724–45. 10.18632/oncotarget.12554 27741508 PMC5356914

[5] Vlachostergios PJ Puca L Beltran H. Emerging variants of castration-resistant prostate cancer. Curr Oncol Rep. (2017) 19:32. 10.1007/s11912-017-0593-6 28361223 PMC5479409

[6] Rowell JL McCarthy DO Alvarez CE. Dog models of naturally occurring cancer. Trends Mol Med. (2011) 17:380–8. 10.1016/j.molmed.2011.02.004 21439907 PMC3130881

[7] Pinho SS Carvalho S Cabral J Reis CA Gartner F. Canine tumors: a spontaneous animal model of human carcinogenesis. Transl Res. (2012) 159:165–72. 10.1016/j.trsl.2011.11.005 22340765

[8] Schrank M Romagnoli S. Prostatic neoplasia in the intact and castrated dog: how dangerous is castration? Animals. (2020) 10:85. 10.3390/ani10010085 31948021 PMC7022700

[9] Cornell KK Bostwick DG Cooley DM Hall G Harvey HJ Hendrick MJ. Clinical and pathologic aspects of spontaneous canine prostate carcinoma: a retrospective analysis of 76 cases. Prostate. (2000) 45:173–83. 10.1002/1097-0045(20001001)45:2<173::AID-PROS12>3.0.CO;2-R 11027417

[10] Ravicini S Baines SJ Taylor A Amores-Fuster I Mason SL Treggiari E. Outcome and prognostic factors in medically treated canine prostatic carcinomas: a multi-institutional study. Vet Comp Oncol. (2018) 16:450–8. 10.1111/vco.12400 29806232

[11] Bennett TC Matz BM Henderson RA Straw RC Liptak JM Selmic LE. Total prostatectomy as a treatment for prostatic carcinoma in 25 dogs. Vet Surg. (2018) 47:367–77. 10.1111/vsu.12768 29400404

[12] Palmieri C Lean FZ Akter SH Romussi S Grieco V. A retrospective analysis of 111 canine prostatic samples: histopathological findings and classification. Res Vet Sci. (2014) 97:568–73. 10.1016/j.rvsc.2014.11.006 25468798

[13] Leroy BE Northrup N. Prostate cancer in dogs: comparative and clinical aspects. Vet J. (2009) 180:149–62. 10.1016/j.tvjl.2008.07.012 18786842

[14] Reimann-Berg N Willenbrock S Murua Escobar H Eberle N Gerhauser I Mischke R. Two new cases of polysomy 13 in canine prostate cancer. Cytogenetic Genome Res. (2011) 132:16–21. 10.1159/000317077 20668368

[15] Eisenloffel C Schmole AC Pews-Davtyan A Brennfuhrer A Kuznetsov SA Hubner R. Interference of a novel indolylmaleimide with microtubules induces mitotic arrest and apoptosis in human progenitor and cancer cells. Biochem Pharmacol. (2013) 85:763–71. 10.1016/j.bcp.2012.12.013 23274302

[16] Kretzschmar C Roolf C Langhammer TS Sekora A Pews-Davtyan A Beller M. The novel arylindolylmaleimide PDA-66 displays pronounced antiproliferative effects in acute lymphoblastic leukemia cells. BMC Cancer. (2014) 14:71. 10.1186/1471-2407-14-71 24502201 PMC3922486

[17] Liu W Beck J Schmidt LC Roolf C Pews-Davtyan A Rutgen BC. Characterization of the novel indolylmaleimides' PDA-66 and PDA-377 effect on canine lymphoma cells. Oncotarget. (2016) 7:35379–89. 10.18632/oncotarget.9297 27177088 PMC5085236

[18] Field JJ Kanakkanthara A Miller JH. Microtubule-targeting agents are clinically successful due to both mitotic and interphase impairment of microtubule function. Bioorganic Med Chem. (2014) 22:5050–9. 10.1016/j.bmc.2014.02.035 24650703

[19] Galluzzi L Vitale I Aaronson SA Abrams JM Adam D Agostinis P. Molecular mechanisms of cell death: recommendations of the nomenclature committee on cell death 2018. Cell Death Differ. (2018) 25:486–541. 10.1038/s41418-018-0102-y 29362479 PMC5864239

[20] Ruan W Lim HH Surana U. Mapping mitotic death: functional integration of mitochondria, spindle assembly checkpoint and apoptosis. Front Cell Dev Biol. (2018) 6:177. 10.3389/fcell.2018.00177 30687704 PMC6335265

[21] Denisenko TV Sorokina IV Gogvadze V Zhivotovsky B. Mitotic catastrophe and cancer drug resistance: a link that must to be broken. Drug Resist Updates. (2016) 24:1–12. 10.1016/j.drup.2015.11.002 26830311

[22] Mc Gee MM. Targeting the mitotic catastrophe signaling pathway in cancer. Mediat Inflamm. (2015) 2015:146282. 10.1155/2015/146282 26491220 PMC4600505

[23] Gardner HL Fenger JM London CA. Dogs as a model for cancer. Ann Rev Anim Biosci. (2016) 4:199–222. 10.1146/annurev-animal-022114-110911 26566160 PMC6314649

[24] Kaighn ME Narayan KS Ohnuki Y Lechner JF Jones LW. Establishment and characterization of a human prostatic carcinoma cell line (PC-3). Invest Urol. (1979) 17:16–23. 447482

[25] Horoszewicz JS Leong SS Kawinski E Karr JP Rosenthal H Chu TM. LNCaP model of human prostatic carcinoma. Cancer Res. (1983) 43:1809–18. 6831420

[26] Winkler S Murua Escobar H Eberle N Reimann-Berg N Nolte I Bullerdiek J. Establishment of a cell line derived from a canine prostate carcinoma with a highly rearranged karyotype. J Heredity. (2005) 96:782–5. 10.1093/jhered/esi085 15994418

[27] Pews-Davtyan A Tillack A Ortinau S Rolfs A Beller M. Efficient palladium-catalyzed synthesis of 3-aryl-4-indolylmaleimides. Organ Biomol Chem. (2008) 6:992–7. 10.1039/b719160j 18327323

[28] Schmole AC Brennfuhrer A Karapetyan G Jaster R Pews-Davtyan A Hubner R. Novel indolylmaleimide acts as GSK-3beta inhibitor in human neural progenitor cells. Bioorgan Med Chem. (2010) 18:6785–95. 10.1016/j.bmc.2010.07.045 20708937

[29] Li H Durbin R. Fast and accurate short read alignment with burrows-Wheeler transform. Bioinformatics. (2009) 25:1754–60. 10.1093/bioinformatics/btp324 19451168 PMC2705234

[30] Luo W Friedman MS Shedden K Hankenson KD Woolf PJ. GAGE: generally applicable gene set enrichment for pathway analysis. BMC Bioinform. (2009) 10:161. 10.1186/1471-2105-10-161 19473525 PMC2696452

[31] Robinson MD McCarthy DJ Smyth GK. edgeR: a bioconductor package for differential expression analysis of digital gene expression data. Bioinformatics. (2010) 26:139–40. 10.1093/bioinformatics/btp616 19910308 PMC2796818

[32] Huang da W Sherman BT Lempicki RA. Systematic and integrative analysis of large gene lists using DAVID bioinformatics resources. Nat Protoc. (2009) 4:44–57. 10.1038/nprot.2008.211 19131956

[33] Huang da W Sherman BT Lempicki RA. Bioinformatics enrichment tools: paths toward the comprehensive functional analysis of large gene lists. Nucleic Acids Res. (2009) 37:1–13. 10.1093/nar/gkn923 19033363 PMC2615629

[34] Sobel RE Sadar MD Cell lines used in prostate cancer research: a compendium of old and new lines–part 2. J Urol. (2005) 173:360–72. 10.1097/01.ju.0000149989.01263.dc 15643173

[35] Cunningham D You Z. *In vitro* and *in vivo* model systems used in prostate cancer research. J Biol Methods. (2015) 2:e17. 10.14440/jbm.2015.63 26146646 PMC4487886

[36] Katsuoka Y Hoshino H Shiramizu M Sakabe K Seiki K. Autoradiographic and cytochemical localization of androgen in human prostatic cancer cell lines. Urology. (1986) 28:228–31. 10.1016/0090-4295(86)90048-8 3489307

[37] Berns EM de Boer W Mulder E. Androgen-dependent growth regulation of and release of specific protein(s) by the androgen receptor containing human prostate tumor cell line LNCaP. Prostate. (1986) 9:247–59. 10.1002/pros.2990090305 2946029

[38] Langeler EG van Uffelen CJ Blankenstein MA van Steenbrugge GJ Mulder E. Effect of culture conditions on androgen sensitivity of the human prostatic cancer cell line LNCaP. Prostate. (1993) 23:213–23. 10.1002/pros.2990230304 7694266

[39] Fork MA Murua Escobar H Soller JT Sterenczak KA Willenbrock S Winkler S. Establishing an *in vivo* model of canine prostate carcinoma using the new cell line CT1258. BMC Cancer. (2008) 8:240. 10.1186/1471-2407-8-240 18706092 PMC2527616

[40] Willenbrock S Wagner S Reimann-Berg N Moulay M Hewicker-Trautwein M Nolte I. Generation and characterisation of a canine EGFP-HMGA2 prostate cancer *in vitro* model. PLoS ONE. (2014) 9:e98788. 10.1371/journal.pone.0098788 24914948 PMC4051699

[41] Liu W Moulay M Willenbrock S Roolf C Junghanss C Ngenazahayo A. Comparative characterization of stem cell marker expression, metabolic activity and resistance to doxorubicin in adherent and spheroid cells derived from the canine prostate adenocarcinoma cell line CT1258. Anticancer Res. (2015) 35:1917–27. 25862843

[42] Packeiser EM Hewicker-Trautwein M Thiemeyer H Mohr A Junginger J Schille JT Characterization of six canine prostate adenocarcinoma and three transitional cell carcinoma cell lines derived from primary tumor tissues as well as metastasis. PLoS ONE. (2020) 15:e0230272 10.1371/journal.pone.0230272 32168360 PMC7069630

[43] Vitale I Galluzzi L Castedo M Kroemer G. Mitotic catastrophe: a mechanism for avoiding genomic instability. Nat Rev Mol Cell Biol. (2011) 12:385–92. 10.1038/nrm3115 21527953

[44] Castedo M Perfettini JL Roumier T Andreau K Medema R Kroemer G. Cell death by mitotic catastrophe: a molecular definition. Oncogene. (2004) 23:2825–37. 10.1038/sj.onc.1207528 15077146

[45] London N Biggins S. Signalling dynamics in the spindle checkpoint response. Nat Rev Mol Cell Biol. (2014) 15:736–47. 10.1038/nrm3888 25303117 PMC4283840

[46] Haschka M Karbon G Fava LL Villunger A. Perturbing mitosis for anti-cancer therapy: is cell death the only answer? EMBO Rep. (2018) 19:e45440. 10.15252/embr.201745440 29459486 PMC5836099

[47] Zhou L Cai X Han X Xu N Chang DC CDK1 switches mitotic arrest to apoptosis by phosphorylating Bcl-2/Bax family proteins during treatment with microtubule interfering agents. Cell Biol Int. (2014) 38:737–46. 10.1002/cbin.10259 24677263

[48] Megyesi J Tarcsafalvi A Seng N Hodeify R Price PM. Cdk2 phosphorylation of Bcl-xL after stress converts it to a pro-apoptotic protein mimicking Bax/Bak. Cell Death Discov. (2016) 2:15066. 10.1038/cddiscovery.2015.66 27226901 PMC4877050

[49] Nakayama Y Inoue T. Antiproliferative fate of the tetraploid formed after mitotic slippage and its promotion; a novel target for cancer therapy based on microtubule poisons. Molecules. (2016) 21:663. 10.3390/molecules21050663 27213315 PMC6274067

[50] Cheng B Crasta K. Consequences of mitotic slippage for antimicrotubule drug therapy. Endocr Relat Cancer. (2017) 24:T97–106. 10.1530/ERC-17-0147 28684541

[51] Goldstein M Kastan MB. Repair vs. checkpoint functions of BRCA1 are differentially regulated by site of chromatin binding. Cancer Res. (2015) 75:2699–707. 10.1158/0008-5472.CAN-15-0400 25939603 PMC4548823

[52] Santaguida S Amon A. Short- and long-term effects of chromosome mis-segregation and aneuploidy. Nat Rev Mol Cell Biol. (2015) 16:473–85. 10.1038/nrm4025 26204159

[53] Mittal K Donthamsetty S Kaur R Yang C Gupta MV Reid MD. Multinucleated polyploidy drives resistance to docetaxel chemotherapy in prostate cancer. Br J Cancer. (2017) 116:1186–94. 10.1038/bjc.2017.78 28334734 PMC5418452

[54] Vail DM MacEwen EG. Spontaneously occurring tumors of companion animals as models for human cancer. Cancer Invest. (2000) 18:781–92. 10.3109/07357900009012210 11107448

[55] Axiak-Bechtel SM Maitz CA Selting KA Bryan JN. Preclinical imaging and treatment of cancer: the use of animal models beyond rodents. Q J Nucl Med Mol Imaging. (2015) 59:303–16. 26200223

[56] Park JS Withers SS Modiano JF Kent MS Chen M Luna JI. Canine cancer immunotherapy studies: linking mouse and human. J Immunother Cancer. (2016) 4:97. 10.1186/s40425-016-0200-7 28031824 PMC5171656

[57] Hansen K Khanna C. Spontaneous and genetically engineered animal models; use in preclinical cancer drug development. Eur J Cancer. (2004) 40:858–80. 10.1016/j.ejca.2003.11.031 15120042

