# Supplementary material for: PDA Indolylmaleimides Induce Anti-Tumor Effects in Prostate Carcinoma Cell Lines Through Mitotic Death
Source: Front Vet Sci. 2021 Jan 20;7:558135. doi: 10.3389/fvets.2020.558135 (PMC7855975; doi:10.3389/fvets.2020.558135)
Supplement: Supplementary file 16 [file Data_Sheet_1.DOCX]

Supplementary Material

**Supplementary Movie 1.** Live cell imaging movie of DMSO-treated PC-3 cells (negative control) over 72 h.

**Supplementary Movie 2.** Live cell imaging movie of PC-3 cells incubated with 0.25 µM PDA-66 over 72 h.

**Supplementary Movie 3.** Live cell imaging movie of PC-3 cells incubated with 0.5 µM PDA-66 over 72 h.

**Supplementary Movie 4.** Live cell imaging movie of PC-3 cells incubated with 1 µM PDA-66 over 72 h.

**Supplementary Movie 5.** Live cell imaging movie of PC-3 cells incubated with 2.5 µM PDA-66 over 72 h.

**Supplementary Movie 6.** Live cell imaging movie of PC-3 cells incubated with 5 µM PDA-66 over 72 h.

**Supplementary Movie 7.** Live cell imaging movie of PC-3 cells incubated with 7.5 µM PDA-66 over 72 h.

**Supplementary Movie 8.** Live cell imaging movie of PC-3 cells incubated with 10 µM PDA-66 over 72 h.

**Supplementary Movie 9.** Live cell imaging movie of DMSO-treated LNCaP cells (negative control) over 72 h.

**Supplementary Movie 10.** Live cell imaging movie of LNCaP cells incubated with 0.25 µM PDA‑66 over 72 h.

**Supplementary Movie 11.** Live cell imaging movie of LNCaP cells incubated with 0.5 µM PDA-66 over 72 h.

**Supplementary Movie 12.** Live cell imaging movie of LNCaP cells incubated with 1 µM PDA-66 over 72 h.

**Supplementary Movie 13.** Live cell imaging movie of LNCaP cells incubated with 2.5 µM PDA-66 over 72 h.

**Supplementary Movie 14.** Live cell imaging movie of LNCaP cells incubated with 5 µM PDA-66 over 72 h.

**Supplementary Movie 15.** Live cell imaging movie of LNCaP cells incubated with 7.5 µM PDA-66 over 72 h.

**Supplementary Movie 16.** Live cell imaging movie of LNCaP cells incubated with 10 µM PDA-66 over 72 h.

**Supplementary Movie 17.** Live cell imaging movie of DMSO-treated CT1258 cells (negative control) over 72 h.

**Supplementary Movie 18.** Live cell imaging movie of CT1258 cells incubated with 0.25 µM PDA‑66 over 72 h.

**Supplementary Movie 19.** Live cell imaging movie of CT1258 cells incubated with 0.5 µM PDA-66 over 72 h.

**Supplementary Movie 20.** Live cell imaging movie of CT1258 cells incubated with 1 µM PDA-66 over 72 h.

**Supplementary Movie 21.** Live cell imaging movie of CT1258 cells incubated with 2.5 µM PDA-66 over 72 h.

**Supplementary Movie 22.** Live cell imaging movie of CT1258 cells incubated with 5 µM PDA-66 over 72 h.

**Supplementary Movie 23.** Live cell imaging movie of CT1258 cells incubated with 7.5 µM PDA-66 over 72 h.

**Supplementary Movie 24.** Live cell imaging movie of CT1258 cells incubated with 10 µM PDA-66 over 72 h.

**Supplementary Movie 25.** Live cell imaging movie of DMSO-treated PC-3 cells (negative control) over 72 h.

**Supplementary Movie 26.** Live cell imaging movie of PC-3 cells incubated with 0.25 µM PDA-377 over 72 h.

**Supplementary Movie 27.** Live cell imaging movie of PC-3 cells incubated with 0.5 µM PDA-377 over 72 h.

**Supplementary Movie 28.** Live cell imaging movie of PC-3 cells incubated with 1 µM PDA-377 over 72 h.

**Supplementary Movie 29.** Live cell imaging movie of PC-3 cells incubated with 2.5 µM PDA-377 over 72 h.

**Supplementary Movie 30.** Live cell imaging movie of PC-3 cells incubated with 5 µM PDA-377 over 72 h.

**Supplementary Movie 31.** Live cell imaging movie of PC-3 cells incubated with 7.5 µM PDA-377 over 72 h.

**Supplementary Movie 32.** Live cell imaging movie of PC-3 cells incubated with 10 µM PDA-377 over 72 h.

**Supplementary Movie 33.** Live cell imaging movie of DMSO-treated LNCaP cells (negative control) over 72 h.

**Supplementary Movie 34.** Live cell imaging movie of LNCaP cells incubated with 0.25 µM PDA‑377 over 72 h.

**Supplementary Movie 35.** Live cell imaging movie of LNCaP cells incubated with 0.5 µM PDA‑377 over 72 h.

**Supplementary Movie 36.** Live cell imaging movie of LNCaP cells incubated with 1 µM PDA-377 over 72 h.

**Supplementary Movie 37.** Live cell imaging movie of LNCaP cells incubated with 2.5 µM PDA‑377 over 72 h.

**Supplementary Movie 38.** Live cell imaging movie of LNCaP cells incubated with 5 µM PDA-377 over 72 h.

**Supplementary Movie 39.** Live cell imaging movie of LNCaP cells incubated with 7.5 µM PDA‑377 over 72 h.

**Supplementary Movie 40.** Live cell imaging movie of LNCaP cells incubated with 10 µM PDA-377 over 72 h.

**Supplementary Movie 41.** Live cell imaging movie of DMSO-treated CT1258 cells (negative control) over 72 h.

**Supplementary Movie 42.** Live cell imaging movie of CT1258 cells incubated with 0.25 µM PDA‑377 over 72 h.

**Supplementary Movie 43.** Live cell imaging movie of CT1258 cells incubated with 0.5 µM PDA‑377 over 72 h.

**Supplementary Movie 44.** Live cell imaging movie of CT1258 cells incubated with 1 µM PDA-377 over 72 h.

**Supplementary Movie 45.** Live cell imaging movie of CT1258 cells incubated with 2.5 µM PDA‑377 over 72 h.

**Supplementary Movie 46.** Live cell imaging movie of CT1258 cells incubated with 5 µM PDA-377 over 72 h.

**Supplementary Movie 47.** Live cell imaging movie of CT1258 cells incubated with 7.5 µM PDA‑377 over 72 h.

**Supplementary Movie 48.** Live cell imaging movie of CT1258 cells incubated with 10 µM PDA‑377 over 72 h.

**
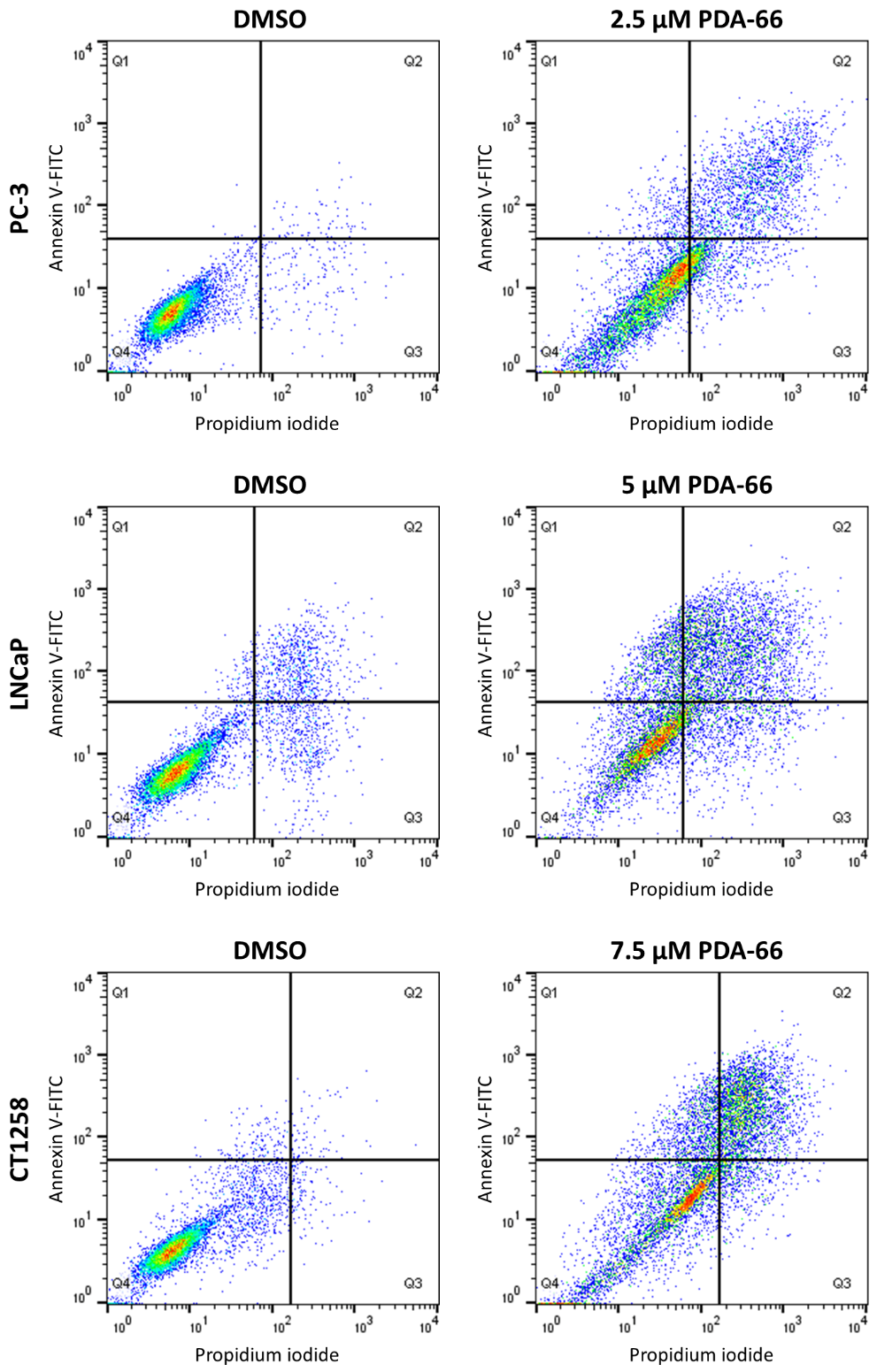
**

**Supplementary Figure 1.** Representative dot blots of flow cytometry data. The three cell lines were stained with Annexin V-FITC and propidium iodide. Cells in area Q4 were counted as vital, cells in Q1 as early apoptotic and cells in Q2 and Q3 as late apoptotic.


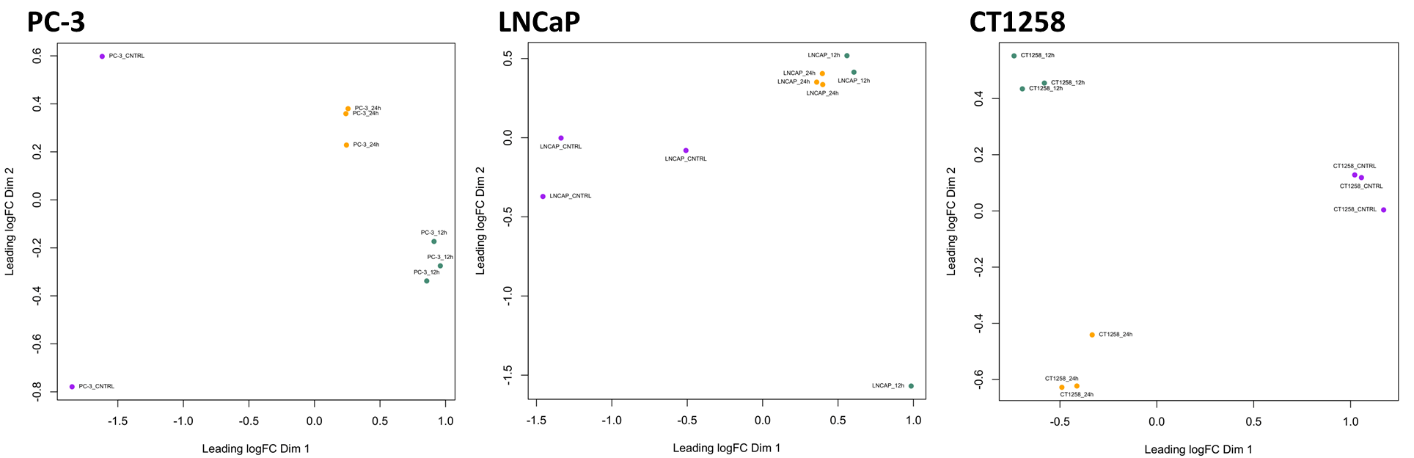


**Supplementary Figure 2.** MDS plots of the RNA-seq data. The distances correspond to the differences in the biological coefficient of variation between the samples of each cell line. The analyzed cells were treated with 15 µM PDA-66 for 12 h and 24 h, and 0.15 % DMSO, respectively.
